# Dishonesty in Testimonials

**Published:** 1917-01-27

**Authors:** 


### The Hospital

The Workers' Newspaper of Administrative Medicine and Institutional Life, Administration, National Insurance and Health.

No. 1599, Vol. LXI. SATURDAY, JANUARY 27, 1917. PRICE ONE PENNY.

### DISHONESTY IN TESTIMONIALS.

It is well known that certain occupations have a demoralising effect upon those who follow them, and render it impossible for their votaries to speak the truth. The selling of horses is a notorious instance, and the promotion of companies is little less so. No one outside of an idiot asylum expects the vendor of a horse to tell the truth about the animal, or the promoter of a company to tell the truth about the venture. If by chance there came on the market a perfect horse, a flawless horse, without spot or blemish, spavin, splint, or vice, the vendor would assuredly declare that the animal had wings. If the company really was an honest venture the promoter might contrive to mar it before he launched it on the market. Nor is this peculiarity confined to serious occupations. The effect of fishing and golf upon the fisherman and the golfer is well enough known. In other walks of life, in other departments of conduct, they may be models of rectitude. They may be honest and upright in business, faithful husbands, wise and affectionate fathers, good citizens, but in matters relating to their favourite recreation they may prove incapable of telling the truth. The moment the topic is introduced away may go their morality, away goes their honesty, away their regard for truth: they scarcely expect to be believed, and certainly no one dreams of believing them.

It has not been sufficiently realised that the writing of testimonials has the same deplorable consequence. A man may be a good man, an honest man, a truthful man in every other capacity in life, but as soon as he sits down to write a testimonial, less than truth flows from his pen like water from a tap. He considers himself as much absolved from the ordinary obligations of morality as the horse-vendor, the company promoter, the golfer, or the fisherman. Of all testimonial-givers the most untruthful and unreliable is the parson. He is so full of charity that he is incapable of justice. He is so anxious to help the lame dog over the stile, to raise up the fallen, and to give the rascal another chance, that he is quite oblivious of the injustice he is perpetrating on the rascal's prospective employer; so he falls from truth. He may, or may not, say what is positively untrue, but he certainly suppresses so much that his document as a whole is false. Worse than this, he will give without hesitation a flaming testimonial to a man of whom he knows very little, and he is apt to assume, in the teeth of centuries of contrary experience, that a regular church-goer must be not only a highly moral person, but a fit and proper person to hold any position that he wants to hold.

This failing of parsons is pretty well known, and no one in his senses accepts the testimonial of a parson as evidence of much more than the weak amiability of the giver. But it is not as well recognised that the testimonials of doctors are almost as worthless. For some reason difficult to discover a man feels himself flattered by the request for a testimonial, and is so well disposed towards his flatterer that he says, not what he knows is true, but what he thinks will please. It is pretty well recognised by the matrons of hospitals and nursing institutions that the testimony of a doctor as to the capacity of a nurse is worth little or nothing. Often the doctor has no real opportunity of judging the capacity of the nurse. As long as she can give a good report and keep up an appearance of efficiency during his brief visits, he is satisfied, though when he is not there to witness she may be lazy, noisy, exacting, and even drunken. Often, too, the element of sex comes in. A pretty woman with an engaging manner can collect favourable testimonials by the dozen, while her plainer but more efficient sister is put off with a few dry words. Reprehensible, no doubt; but while doctors are men and nurses are women such things will occur. Age, position, and eminence of the testimonial-giver go for much: they should go for nothing; and in the estimation of the experienced they do go for nothing. The only things that really count are the opportunities that the witness has of knowing and his capability and willingness to judge impartially, and testify carefully and truthfully. These are the only things that count in the giver of a testimonial, but how is the reader of the document to judge of them? Obviously he cannot; and the only safe ground to go upon is the whole past record of those whom he thinks of employing—either this, or that penetrating insight into character which but few people possess. To such people testimonials are useless: to others they are usually misleading.

